# Outcomes of Allogeneic Hematopoietic Stem Cell Transplantation in Adult Acute Myeloid Leukemia: A Systematic Review

**DOI:** 10.7759/cureus.92549

**Published:** 2025-09-17

**Authors:** Alaa Babiker Mohamed Ahmed, Ehab A Elagab, Eilaf Abuelgasim Abdalla Ahmed, Nada Eltigani Hassan Mahgoub, Fatima Suliman Dawod Faky, Nada Abdelrahman Shamina, Nagla Subahi

**Affiliations:** 1 Internal Medicine, Shaikh Khalifa General Hospital, Umm Al Quwain, ARE; 2 Department of Pathology, College of Medicine, Najran University, Najran, SAU; 3 Internal Medicine, King Saud Hospital, Al Qassim, SAU; 4 Hematology, University Hospital Kerry, Tralee, IRL; 5 Clinical Pathology, Hafar Al Batin Central Hospital, Hafar Al Batin, SAU; 6 Chemical Pathology, Nottingham City Hospital, Nottingham, GBR; 7 Hematology, Prince Mohammad Bin Nasser Hospital, Jazan, SAU

**Keywords:** acute myeloid leukemia, allogeneic hematopoietic stem cell transplantation, non-relapse mortality, prognostic factors, relapse, survival outcomes

## Abstract

Allogeneic hematopoietic stem cell transplantation (allo-HSCT) is a potentially curative treatment for adult acute myeloid leukemia (AML), but its outcomes are influenced by donor selection, conditioning regimens, and patient-specific factors. This systematic review synthesizes recent evidence on survival, relapse, non-relapse mortality (NRM), and prognostic factors to guide clinical decision-making. A comprehensive search of PubMed, Scopus, Web of Science, Embase, and ClinicalTrials.gov (January 2020-August 2025) identified 10 studies meeting the inclusion criteria. Studies were evaluated for outcomes (overall survival (OS), disease-free survival (DFS), relapse, and NRM) and risk of bias using the Newcastle-Ottawa Scale (NOS) and Cochrane Risk of Bias (RoB) 2 tool. Haploidentical HSCT improved one-year survival (77.9% vs. 62.0% with chemotherapy) in elderly AML, with lower relapse (16.5% vs. 56.6%). Matched sibling donors (MSD) yielded superior two-year OS (62.4%) compared to unrelated/haploidentical donors, though relapse rates were comparable. Reduced-intensity conditioning (RIC) increased NRM in patients ≥70 years, while nonmyeloablative regimens improved tolerability. Transplantation in complete remission (CR) was critical (five-year OS: 58% vs. 6% for non-CR). Autologous HSCT outperformed allo-HSCT in acute promyelocytic leukemia (APL) complete remission 2 (CR2) (82.4% vs. 64.3% two-year OS). Allo-HSCT benefits intermediate/poor-risk AML, with MSD and CR transplantation offering optimal outcomes. Haploidentical HSCT is viable for older patients lacking matched donors. Future research should prioritize prospective trials to refine donor selection and conditioning strategies.

## Introduction and background

Acute myeloid leukemia (AML) is an aggressive and heterogeneous blood cancer characterized by uncontrolled proliferation of immature myeloid cells in the bone marrow and blood [1]. It is the most common acute leukemia in adults, with incidence rising sharply with age, and it continues to carry a high risk of relapse and death despite progress in chemotherapy and supportive care [2]. Prognosis remains variable and depends on genetic alterations, patient age, performance status, and comorbidities [3].

Allogeneic hematopoietic stem cell transplantation (allo-HSCT) is the most effective curative strategy for adults with AML, particularly those in intermediate- and poor-risk groups [4]. Its benefit stems largely from the graft-versus-leukemia effect, whereby donor immune cells eliminate residual malignant cells [5]. However, transplantation is associated with substantial risks - including graft-versus-host disease (GVHD), infections, and treatment-related mortality - that may compromise outcomes, especially in older or medically vulnerable patients [6].

Advances in the past two decades have improved the safety and applicability of transplantation. Reduced-intensity conditioning (RIC) regimens, better donor matching strategies (including haploidentical donors), and enhanced supportive care have widened eligibility [7]. In parallel, molecular profiling now helps refine patient selection and predict benefit. Nevertheless, uncertainties persist. Outcomes differ by donor type, with matched sibling donors (MSD) often superior, though haploidentical options show promise in older patients. Remission status at transplant remains a critical determinant of survival, and specific subtypes such as acute promyelocytic leukemia (APL) may follow different treatment pathways, including autologous transplantation [8].

Given these complexities, there is a clear need to consolidate current evidence. This systematic review evaluates outcomes of allo-HSCT in adult AML, focusing on overall survival (OS), disease-free survival (DFS), relapse, non-relapse mortality (NRM), and prognostic factors. By synthesizing findings from the past decade, it aims to provide clinicians with an accessible and up-to-date perspective on the role of allo-HSCT in AML management and to highlight ongoing challenges in donor availability, conditioning strategies, and global access to transplantation.

## Review

Methods

Eligibility Criteria

This systematic review included studies that evaluated the outcomes of allo-HSCT in adult patients (≥18 years) with AML. Studies were eligible if they reported on OS, DFS, relapse incidence, NRM, or GVHD following allo-HSCT. Both prospective and retrospective cohort studies, registry analyses, and interventional trials were considered. Studies focusing exclusively on pediatric populations, autologous transplantation, or secondary AML were excluded. Additionally, non-English articles, reviews, editorials, conference abstracts without full data, and studies with incomplete outcome reporting were excluded. Studies published before January 2020 were also excluded.

Information Sources

A comprehensive literature search was conducted on PubMed, Scopus, Web of Science, Embase, and ClinicalTrials.gov to identify relevant studies published from January 2020 to August 2025. This approach was adopted to ensure the inclusion of the most relevant studies from the last five years. The databases were selected to ensure broad coverage of peer-reviewed biomedical literature, clinical trial registries, and multidisciplinary sources, capturing both published and ongoing research relevant to allo-HSCT in adult AML.

Search Strategy

The search strategy combined Medical Subject Headings (MeSH) and free-text terms related to “acute myeloid leukemia,” “allogeneic hematopoietic stem cell transplantation,” “outcomes,” “survival,” and “relapse.” Boolean operators and database-specific filters were applied to maximize sensitivity while restricting results to adult populations and human studies. The full search strategies for all databases are provided in the supplementary materials.

Selection Process

All retrieved citations were imported into EndNote software for management, and duplicates were systematically removed. Titles and abstracts were screened independently by two reviewers to assess eligibility. Full texts of potentially relevant articles were then reviewed to confirm inclusion. Discrepancies were resolved through discussion, and if necessary, a third reviewer was consulted to reach a consensus.

Data Collection Process

Data extraction was conducted using a standardized form developed for this review. Extracted data included study characteristics (author, year, country, design), patient demographics, AML risk classification, transplant details (donor type, conditioning regimen), follow-up duration, and reported outcomes including OS, DFS, relapse, NRM, and GVHD incidence. Two reviewers independently performed data extraction, and any disagreements were reconciled through discussion to ensure accuracy and completeness.

Risk of Bias Assessment

The quality and risk of bias of included studies were assessed using the Newcastle-Ottawa Scale (NOS) [9] for observational studies and the Cochrane Risk of Bias 2 (RoB 2) tool [10] for randomized controlled trials. The NOS evaluates selection, comparability, and outcome domains, while RoB 2 assesses biases arising from randomization, deviations from intended interventions, missing data, outcome measurement, and selective reporting. Two reviewers independently performed risk of bias assessments, with discrepancies resolved through discussion.

Data Synthesis

Given the substantial clinical heterogeneity among studies in terms of patient populations, AML risk stratification, conditioning regimens, donor types, follow-up durations, and outcome definitions, a quantitative meta-analysis was not performed. Instead, a qualitative synthesis of study findings was undertaken to summarize the evidence on survival, relapse, NRM, and prognostic factors influencing allo-HSCT outcomes in adult AML. Outcomes were reported narratively and in tabular form to allow meaningful comparisons across studies.

Reporting

The review was conducted and reported in accordance with the PRISMA (Preferred Reporting Items for Systematic reviews and Meta-Analyses) 2020 guidelines to ensure transparency, reproducibility, and methodological rigor [11]. A PRISMA flow diagram detailing the study selection process is provided to illustrate the number of records identified, screened, excluded, and included in the review.

Results

Study Selection Process

**Figure 1:**
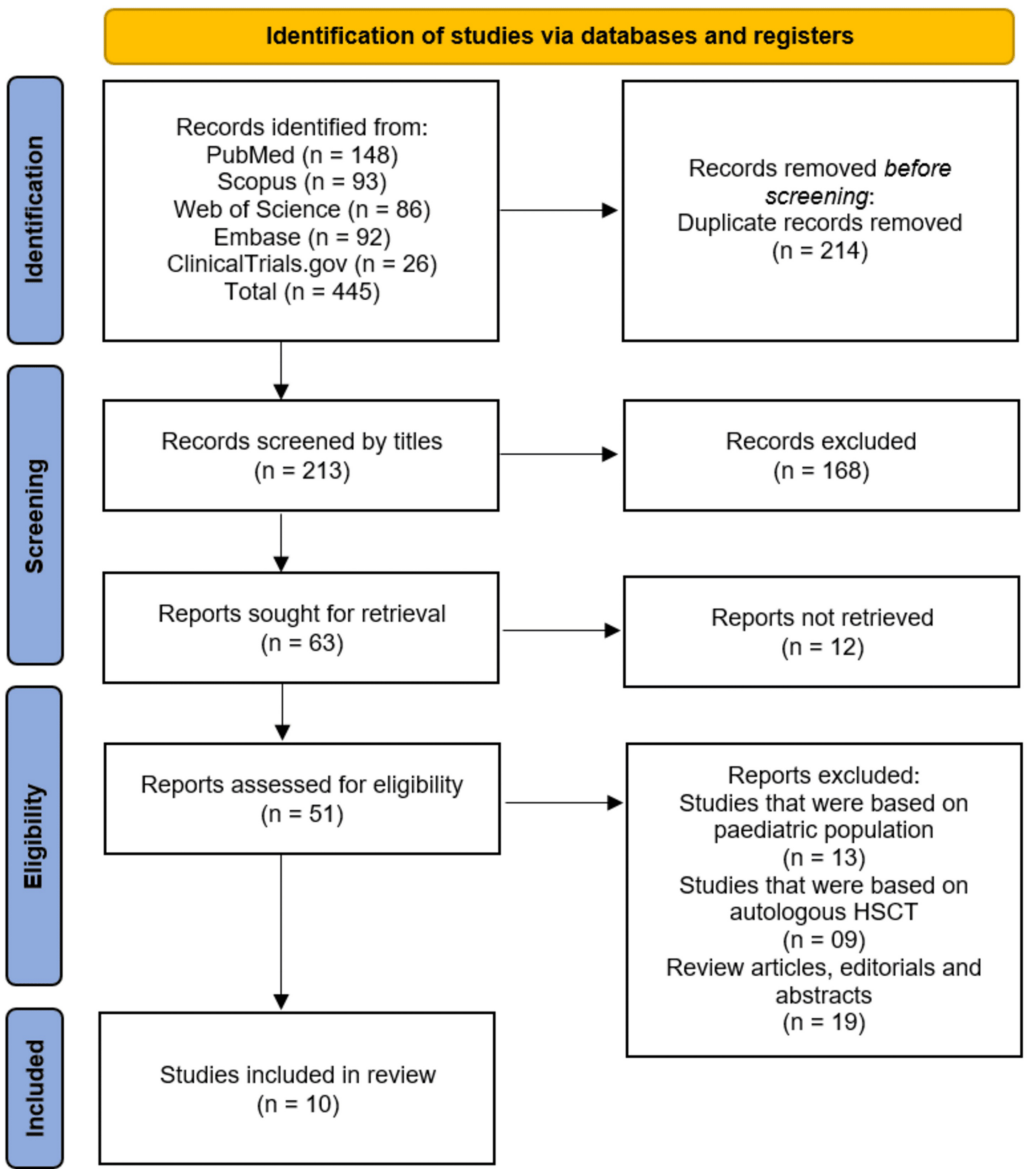
PRISMA flowchart illustrating the study selection process PRISMA: Preferred Reporting Items for Systematic reviews and Meta-Analyses

The study selection process followed the PRISMA guidelines and is summarized in the flow diagram (Figure 1). A total of 445 records were identified through systematic searches of PubMed (n=148), Scopus (n=93), Web of Science (n=86), Embase (n=92), and ClinicalTrials.gov (n=26). After removing 214 duplicate records, 213 studies underwent title screening, of which 168 were excluded for irrelevance. The remaining 63 full-text articles were sought for retrieval, with 12 unavailable, leaving 51 reports assessed for eligibility. Of these, 13 studies were excluded for focusing on pediatric populations, nine for evaluating autologous HSCT, and 19 for being review articles, editorials, or abstracts. Ultimately, 10 studies [12-21] met the inclusion criteria and were included in the systematic review.

Characteristics of Included Studies

The systematic review included 10 studies [12-21] investigating the outcomes of allo-HSCT in adult AML patients. The studies were conducted across diverse regions, including China, the USA, Europe, Japan, South Korea, and India, and encompassed various study designs such as retrospective cohort analyses, randomized controlled trials, and registry-based studies (Table 1). Sample sizes ranged from 22 to 701 patients, with a focus on older adults (≥55 years) in most studies [12-17]. Donor types included MSD, matched unrelated donor (MUD), Haplo, and cord blood, with conditioning RIC or NMA for older patients [12-15,17]. Follow-up durations varied from one year to a median of 5.4 years, with outcomes reported for OS, leukemia-free survival (LFS), relapse, and NRM.

**Table 1:** Characteristics of included studies MRD: matched related donor; MUD: matched unrelated donor; Haplo: haploidentical donor; Cord: umbilical cord blood; AML: acute myeloid leukemia; APL: acute promyelocytic leukemia; RIC: reduced-intensity conditioning; OS: overall survival; LFS: leukemia-free survival; NRM: non-relapse mortality; NMA: nonmyeloablative; CR1: first complete remission; HLA: human leukocyte antigen; TBI: total body irradiation; GVHD: graft-versus-host disease; ALWP-EBMT: Acute Leukemia Working Party of the European Society for Blood and Marrow Transplantation; MSD: matched sibling donor; PB: peripheral blood; IQR: interquartile range

Study	Country/region	Study design	Sample size (n)	Patient population (age, disease status)	Donor type (MRD, MUD, Haplo, Cord)	Conditioning regimen	Follow-up duration
Li et al. (2025) [12]	China	Retrospective, case-controlled (propensity score matched)	307 total (71 Haplo-HCT, 236 Chemo; 69 pairs after matching)	Adult AML patients, ≥55 years old, in complete remission	Haploidentical donors (Haplo)	72.5% received RIC	1-year outcomes reported (OS, LFS, relapse, NRM)
Vo et al. (2025) [13]	USA (single-institution study)	Retrospective cohort study	495	Adults with AML in remission; age groups: 60–64 (n=184), 65–69 (n=189), ≥70 (n=122)	NR	RIC and NMA conditioning	2006–2023
Niederwieser et al. (2024) [14]	25 trial sites in Germany, the Netherlands, Switzerland, France, and Australia	Randomized controlled trial (HCT vs. non-HCT)	245 registered; 179 eligible; 125 randomized (HCT n=83, non-HCT n=42)	Adults aged 60–75 years, AML in CR1	HLA-identical matched donors (MRD/MUD) in 75.4% of patients	Fludarabine + low-dose TBI; cyclosporine + mycophenolate mofetil for GVHD prophylaxis	Up to 5 years
Maffini et al. (2024) [15]	Europe (ALWP-EBMT database)	Retrospective cohort analysis (2010–2021)	360	Adults ≥70 years (median 72, range 70–79); AML not in remission	MSD (n=58), 10/10 MUD (n=228), Haplo (n=74); graft source PB in 92%, marrow more frequent in Haplo	NR	Median 35.5 months
Socie et al. (2023) [16]	France	Single-center retrospective cohort	NR	All adult AML patients at diagnosis, irrespective of age and comorbidities; age range: 16–70+ years	MRD, MUD, Haplo (broad donor access mentioned)	NR	Until the last follow-up
Maffini et al. (2023) [17]	Multicenter	Retrospective cohort/registry analysis	701	Adults ≥70 years, AML in first CR1	MSD, 10/10 MUD, 9/10 mUD, Haplo	NR	2 years (outcomes reported at 2 years)
Yanada et al. (2022) [18]	Japan (nationwide registry)	Retrospective cohort study (registry-based)	195	Adults ≥16 years with relapsed APL; subgroups: in CR vs. non-CR, prior autologous HCT vs. no prior autologous HCT	Not specified in abstract (likely MRD, MUD, Haplo, Cord, but not detailed)	NR	Median 5.4 years for survivors
Min et al. (2022) [19]	South Korea (Catholic Hematology Hospital)	Retrospective cohort study (2000–2019)	52 relapsed APL patients	Adult patients with relapsed APL (age not specified in abstract)	NR	NR	NR
Sanz et al. (2021) [20]	Europe (EBMT registry, multicenter)	Retrospective cohort study (registry-based)	228 (allo-HSCT group, compared with 341 auto-HSCT)	Adult patients with APL in CR2	NR	NR	Median follow-up (outcomes reported at 2 years)
Kulkarni et al. (2020) [21]	India	Open-label, nonrandomized, phase II	22	Adults, relapsed APL; median age 26.5 years (IQR 17.5–41.5)	Autologous (12 patients underwent auto-SCT); the rest received maintenance therapy	NR	Median 48 months (range 28–56.3)

Outcomes of Allogeneic HSCT

The outcomes of allo-HSCT varied by donor type, conditioning intensity, and patient age. Li et al. [12] demonstrated superior one-year OS (77.9% vs. 62.0%) and LFS (74.1% vs. 42.0%) with haploidentical HSCT compared to chemotherapy in AML patients ≥55 years, alongside significantly lower relapse rates (16.5% vs. 56.6%) and acceptable NRM (9.4%). Similarly, Vo et al. [13] reported that allo-HSCT was feasible in patients ≥60 years, though those ≥70 years had shorter OS and higher NRM with RIC, while NMA mitigated age-related risks. Niederwieser et al. [14] found that allo-HSCT reduced relapse (37.8% vs. 91.1%) and improved LFS (24.5 vs. 15.6 months) compared to chemotherapy in patients aged 60-75 years, albeit with higher NRM (33.4%) that offset OS benefits.

Donor type significantly influenced outcomes. Maffini et al. [15,17] observed superior OS and LFS with MSD (OS: 62.4%; LFS: 47.6%) compared to MUD (OS: 43%; LFS: 37.5%) and Haplo (OS: 25.9%; LFS: 26.5%) in older patients, attributed to lower NRM in MSD recipients (17.5% vs. 32.2% for MUD and 43.9% for Haplo). However, Haplo and MUD were associated with lower relapse rates in some cohorts [17]. For relapsed AML, Yanada et al. [18] noted five-year OS of 58% in patients transplanted in complete remission (CR), while non-CR status and prior autologous HSCT predicted poorer outcomes (OS: 6%). Auto-HSCT outperformed allo-HSCT in APL CR2 (two-year OS: 82.4% vs. 64.3%), driven by lower NRM (2.7% vs. 17.3%) (Table 2) [20].

**Table 2:** Outcomes of allogeneic HSCT in adult AML HSCT: hematopoietic stem cell transplantation; AML: acute myeloid leukemia; DFS: disease-free survival; RFS: relapse-free survival; NRM: non-relapse mortality; Haplo: haploidentical donor; OS: overall survival; LFS: leukemia-free survival; RIC: reduced-intensity conditioning; MSD: matched sibling donor; MUD: matched unrelated donor; CR1: first complete remission; APL: acute promyelocytic leukemia

Study	OS (%) at 2/5 years	DFS/RFS (%)	Relapse rate (%)	NRM (%)	Key findings
Li et al. (2025)[12]	1-year OS: 77.9% (Haplo) vs. 62.0% (Chemo) (no 2/5-year data provided)	1-year LFS: 74.1% (Haplo) vs. 42.0% (Chemo)	1-year relapse: 16.5% (Haplo) vs. 56.6% (Chemo)	1-year NRM: 9.4% (Haplo)	Haplo-HSCT significantly improved OS and LFS compared to chemotherapy, with markedly reduced relapse incidence. NRM was acceptable. Suggests Haplo-HSCT as a beneficial option for AML patients ≥55 years
Vo et al. (2025) [13]	Not directly reported; OS in ≥70 yrs was slightly shorter but not statistically significant (p=0.11). With RIC, ≥70 years had significantly shorter OS (HR=3.46, p<0.001)	No significant differences in RFS among age groups overall. With RIC, ≥70 years had shorter RFS (HR=3.79, p<0.001)	No significant differences overall. With RIC, ≥70 years had a higher relapse risk (HR=3.47, p=0.012)	Higher NRM in ≥70 years vs. 60–64 years (p=0.022). With RIC, ≥70 years had higher NRM (HR=3.88, p=0.001).	Allo-HSCT feasible ≥70 yrs; RIC ↑relapse/NRM, ↓OS/RFS; NMA reduces age effect; 60–69 years: RIC ↓relapse, ↑NRM
Niederwieser et al. (2024) [14]	5-year RM-OS: 27.8 months (HCT) vs. 28.6 months (non-HCT) → no significant OS difference	5-year RM-LFS: 24.5 months (HCT) vs. 15.6 months (non-HCT), p=0.022	5-year relapse: 37.8% (HCT) vs. 91.1% (non-HCT), p<0.0001	5-year NRM: 33.4% (HCT) vs. 0% (non-HCT)	HCT significantly reduced relapse and improved LFS compared to chemotherapy, but did not improve overall survival due to higher NRM
Maffini et al. (2024) [15]	MSD: 62.4% (2y), MUD: 43%, Haplo: 25.9%	MSD: 47.6%, MUD: 37.5%, Haplo: 26.5%	MSD: 34.9%, MUD: 30.2%, Haplo: 29.6%	MSD: 17.5%, MUD: 32.2%, Haplo: 43.9%	MSD showed superior OS and DFS due to lower NRM; MUD and Haplo associated with inferior outcomes, with Haplo worst despite slightly lower relapse
Socie et al. (2023) [16]	NR (HSCT improved OS in intermediate- and poor-risk patients, HR=0.51; p=0.004)	NR	NR	NR	HSCT improved OS in intermediate/poor-risk AML; limited in good-risk. 4-year incidence 21.9%; barriers: comorbidities, remission, donor; Haplo expansion may increase use
Maffini et al., (2023) [17]	2-year OS: 48.1%	2-year LFS: 45.3%	25.2%	29.5%	Allo-HSCT in AML patients ≥70 years in CR1 is feasible with acceptable outcomes. Haplo and MUD had lower relapse rates compared to MSD. Haplo showed prolonged LFS. MUD was associated with the highest NRM. GRFS reported at 33.4%
Yanada et al. (2022) [18]	5-year OS: 58% (CR, no prior auto-HCT), 39% (non-CR, no prior auto-HCT), 47% (CR with prior auto-HCT), 6% (non-CR with prior auto-HCT)	NR	NR	NR	Allo-HCT offers long-term survival in relapsed APL. Poor outcomes for patients transplanted in non-CR and those with prior auto-HCT. Non-CR status and prior auto-HCT independently predicted worse OS
Min et al. (2022) [19]	NR (6/7 alive after salvage allo-HSCT)	NR	NR	NR	Allogeneic HSCT was used mainly as salvage therapy after second relapse; among 7 patients treated, 6 survived. Outcomes after allo-HSCT were favorable compared to ATO without HSCT. No significant difference in OS across post-remission treatments, but allo-HSCT appeared effective in second relapse
Sanz et al. (2021) [20]	2-yr OS: 64.3% (allo-HSCT) vs. 82.4% (autoHSCT)	2-yr LFS (DFS): 54.7% (allo-HSCT) vs. 74.5% (auto-HSCT)	28% (allo-HSCT) vs. 22.9% (auto-HSCT); NS (p=0.28)	17.3% (allo-HSCT) vs. 2.7% (auto-HSCT)	Auto-HSCT had superior OS and LFS compared to allo-HSCT in APL CR2, mainly due to lower NRM. Relapse rates were similar between groups. Older age and shorter time from diagnosis to transplant were associated with worse outcomes
Kulkarni et al. (2020) [21]	95.5% (auto-SCT subgroup, median follow-up 48 months)	95.5% (auto-SCT subgroup)	3/22 (13.6%) overall; 1/12 (8.3%) in auto-SCT group	NR	Addition of bortezomib to ATO-based salvage therapy in relapsed APL is safe and effective; patients undergoing auto-SCT in molecular remission had excellent OS and RFS; the maintenance therapy group had higher relapse

Prognostic Factors Influencing Outcomes

Key prognostic factors included donor type, conditioning intensity, disease status, and age (Table 3). Haploidentical HSCT was associated with reduced relapse and improved survival in elderly AML [12], while MSD remained optimal for older patients due to lower NRM [15,17]. Age ≥70 years predicted higher NRM and relapse with RIC, though NMA improved tolerability [13]. Disease status at transplantation was critical; non-CR and prior autologous HSCT significantly worsened OS in relapsed AML [18,19]. For APL, auto-HSCT in molecular remission yielded excellent outcomes (95.5% OS) [21], whereas allo-HSCT was reserved for salvage therapy in second relapse [19].

**Table 3:** Prognostic factors influencing outcomes after allogeneic HSCT in adult AML HSCT: hematopoietic stem cell transplantation; AML: acute myeloid leukemia; OS: overall survival; DFS: disease-free survival; NRM: non-relapse mortality; TRM: treatment-related mortality; LFS: leukemia-free survival; RIC: reduced-intensity conditioning; NMA: nonmyeloablative; MSD: matched sibling donor; MUD: matched unrelated donor

Study	Prognostic factor assessed	Effect on OS/DFS	Effect on relapse	Effect on NRM/TRM	Clinical interpretation
Li et al. (2025) [12]	Treatment type: Haplo HSCT vs. chemotherapy in elderly AML patients (≥55 years)	Haplo HSCT significantly improved 1-year OS (77.9% vs. 62.0%) and LFS (74.1% vs. 42.0%) compared to chemotherapy	Haplo HSCT significantly reduced relapse incidence (16.5% vs. 56.6%)	1-year NRM in the Haplo HSCT group was 9.4%	Haplo HSCT provides a substantial survival advantage over chemotherapy, mainly by lowering relapse risk with acceptable NRM, making it a favorable option for eligible elderly AML patients
Vo et al. (2025) [13]	Age and conditioning Intensity	Age ≥70 had shorter OS/RFS and higher relapse/NRM with RIC, but no adverse effect with NMA; age 65–69 outcomes similar to 60–64	RIC lowered relapse in 60–69 yrs but not ≥70 yrs	RIC increased NRM across all age groups	NMA is preferable in ≥70 yrs, while RIC may benefit 60–69 yrs; transplant feasible across older adults with tailored conditioning
Niederwieser et al. (2024) [14]	Allo-HSCT vs. non-HSCT in elderly AML patients (60–75 years) in CR1	DFS (RM-LFS) significantly improved with HSCT (24.5 vs. 15.6 months; p=0.022). OS showed no significant difference (27.8 vs. 28.6 months)	Relapse rate markedly reduced with HSCT (37.8% vs. 91.1%; p<0.0001)	NRM substantially higher in the HSCT group (33.4% vs. 0%)	HSCT improves disease control by lowering relapse and extending leukemia-free survival, but higher non-relapse mortality offsets OS benefit in older patients
Maffini et al. (2024) [15]	Donor type (MSD vs. MUD vs. Haplo)	MSD showed superior OS (62.4%) and DFS (47.6%) compared to MUD (OS 43%, DFS 37.5%) and Haplo (OS 25.9%, DFS 26.5%); Haplo and MUD were associated with inferior survival in multivariate analysis	Relapse rates were similar across donor types (MSD 34.9%, MUD 30.2%, Haplo 29.6%)	NRM lowest in MSD (17.5%), higher in MUD (32.2%) and Haplo (43.9%); Haplo and MUD significantly increased risk vs. MSD	MSD remains the optimal donor choice in older AML with active disease due to better survival and lower NRM, while Haplo and MUD carry higher mortality despite comparable relapse rates
Socie et al. (2023) [16]	ELN risk (intermediate/poor vs. good), age, eligibility & donor availability	HSCT improved OS in intermediate/poor-risk AML (HR=0.51; p=0.004), benefit less clear in good-risk; younger patients had better access and outcomes; older/comorbid patients rarely transplanted	Relapse reduction implied in intermediate/poor-risk groups	NR	HSCT offers survival benefit mainly in intermediate/poor-risk and younger AML patients; age, comorbidities, and donor availability remain key barriers
Maffini et al. (2023) [17]	Donor type (MSD vs. Haplo, UD, mUD) in AML patients ≥70 years (CR1)	Haplo improved LFS; overall 2-yr OS 48.1%, LFS 45.3%	Haplo and UD reduced relapse (HR 0.46 and 0.44)	mUD increased NRM (HR 2.33); overall NRM 29.5%	Allo-HSCT feasible in selected ≥70 yrs; Haplo/UD favorable for relapse, mUD linked to higher mortality
Yanada et al. (2022) [18]	Non-CR at transplantation and prior autologous HCT	Both factors significantly worsened OS (HR 1.74 for non-CR; HR 2.10 for prior auto-HCT). 5-yr OS: 58% (CR, no prior auto-HCT) vs. 39% (non-CR) and 47% (CR after prior auto-HCT) vs. 6% (non-CR after prior auto-HCT)	Higher relapse risk implied in non-CR and prior auto-HCT groups (not directly quantified)	NR	Non-CR status and prior autologous HCT are adverse prognostic factors, with extremely poor outcomes when both are present
Min et al. (2022) [19]	Allo-HSCT in relapsed APL (post-ATO reinduction and as salvage in second relapse)	No significant survival benefit compared to ATO/auto-HSCT in first relapse; improved survival as salvage in second relapse	High relapse after ATO/auto-HSCT (≈50%); allo-HSCT reduced relapse-related mortality in the second relapse	NR	Allo-HSCT has limited benefit in first relapse but is effective as salvage therapy in second relapse, improving survival outcomes
Sanz et al. (2021) [20]	Allo-HSCT vs. Auto-HSCT, older age, shorter time from diagnosis to transplant	Worse OS/LFS with alloHSCT, older age, and shorter interval	No significant difference	Higher NRM with alloHSCT	Auto-HSCT led to better survival mainly due to lower NRM; older age and shorter time to transplant negatively impacted outcomes
Kulkarni et al. (2020) [21]	Post-remission therapy (auto-SCT vs. maintenance) and molecular remission at the end of induction	Auto-SCT in molecular remission: high OS/DFS (all except 1 relapse-free at 48 months)	Auto-SCT: 1 relapse; maintenance: 3 relapses	Minimal NRM; 1 therapy discontinuation due to neuropathy	Auto-SCT in molecular remission is associated with superior survival and lower relapse; addition of bortezomib to ATO is safe

ELN risk stratification highlighted the survival benefit of allo-HSCT in intermediate/poor-risk AML (HR=0.51) but limited utility in good-risk disease [16]. Older age and shorter time from diagnosis to transplant negatively impacted outcomes [20], underscoring the importance of timely transplantation and careful patient selection.

Risk of Bias Findings

The risk of bias assessment, conducted using the NOS for cohort studies and the Cochrane RoB 2 tool for RCTs, revealed that most studies had a low to moderate risk of bias. The RCT by Niederwieser et al. [14] was rated as low risk across all domains of the RoB 2 tool, including randomization, deviations, missing data, outcome measurement, and selective reporting. Among the cohort studies, Li et al. [12] achieved a perfect NOS score (9/9), indicating minimal bias, while Vo et al. [13], Maffini et al. [15,17], Socie et al. [16], and Sanz et al. [20] were also rated as low risk (7/9) due to robust selection, comparability, and outcome assessment. However, Yanada et al. [18] exhibited moderate risk (5/9) owing to incomplete adjustment for confounders and missing donor details, whereas Min et al. [19] and Kulkarni et al. [21] were deemed high risk (4/9) due to small sample sizes, lack of control groups, and insufficient outcome data (Tables 4, 5).

**Table 4:** Risk of bias using NOS tool NOS: Newcastle-Ottawa Scale

Study	Selection (representativeness, exposure ascertainment, no baseline disease, follow-up start)	Comparability (adjustment for confounders)	Outcome (assessment, follow-up adequacy, completeness)	Total score	Risk of bias
Li et al. (2025) [12]	★★★★	★★	★★★	9/9	Low
Vo et al. (2025) [13]	★★★	★	★★★	7/9	Low
Maffini et al. (2024) [15]	★★★	★★	★★	7/9	Low
Socie et al. (2023) [16]	★★★	★★	★★	7/9	Low
Maffini et al. (2023) [17]	★★★	★	★★★	7/9	Low
Yanada et al. (2022) [18]	★★	★	★★	5/9	Moderate
Min et al. (2022) [19]	★★	★	★	4/9	High
Sanz et al. (2021) [20]	★★★	★	★★★	7/9	Low
Kulkarni et al. (2020) [21]	★★	★	★	4/9	High

**Table 5:** Risk of bias assessment for RCT using Cochrane RoB 2 tool RCT: randomized controlled trial

Study	Randomization	Deviations from intended interventions	Missing data	Outcome measurement	Selective reporting	Overall bias
Niederwieser et al. (2024) [14]	Low	Low	Low	Low	Low	Low

Discussion

The findings of this systematic review provide a comprehensive synthesis of the current evidence regarding outcomes and prognostic factors associated with allo-HSCT in adult AML. The review included 10 studies encompassing diverse populations, donor types, and conditioning regimens, offering valuable insights into the efficacy, safety, and clinical applicability of allo-HSCT across different patient subgroups. The results highlight the critical role of donor selection, conditioning intensity, disease status, and patient age in determining transplantation outcomes, while also underscoring the trade-offs between relapse reduction and NRM.

One of the most compelling findings was the superiority of haploidentical HSCT over chemotherapy in elderly AML patients (≥55 years), as demonstrated by Li et al. [12]. The study reported significantly improved one-year OS (77.9% vs. 62.0%) and LFS (74.1% vs. 42.0%) alongside a marked reduction in relapse rates (16.5% vs. 56.6%). These results suggest that haploidentical HSCT may be a viable alternative for older patients who lack matched donors, a finding consistent with recent literature advocating for the expansion of donor pools to include haploidentical options [22]. However, the study’s focus on short-term outcomes (one-year follow-up) necessitates caution, as long-term survival and late complications were not assessed. This limitation is particularly relevant given that older patients often face competing risks beyond the first year post-transplant, such as chronic GVHD and secondary malignancies [23].

The impact of age and conditioning intensity on transplantation outcomes was further elucidated by Vo et al. [13], who reported that while allo-HSCT was feasible in patients ≥60 years, those aged ≥70 experienced shorter OS and higher NRM with RIC. Notably, NMA regimens mitigated age-related risks, suggesting that conditioning intensity should be tailored to individual patient frailty and comorbidities. These findings align with prior studies emphasizing the importance of personalized conditioning approaches in older adults [24]. However, the study’s retrospective design and single-center nature may limit generalizability, as institutional practices and patient selection biases could influence outcomes.

Donor type emerged as a pivotal prognostic factor, with MSD consistently associated with superior survival and lower NRM compared to MUD and haploidentical donors [15,17]. Maffini et al. [15] reported two-year OS rates of 62.4% for MSD recipients versus 43% for MUD and 25.9% for haploidentical donors, underscoring the enduring advantage of MSD in older AML patients. These observations are supported by meta-analyses demonstrating that MSD transplants yield better outcomes due to lower incidences of severe GVHD and NRM [25]. However, the comparable relapse rates across donor types [15,17] suggest that haploidentical and MUD transplants may still offer disease control benefits, particularly for high-risk patients without MSD options. This dichotomy highlights the need for risk-adapted donor selection algorithms that balance relapse prevention against transplantation-related mortality.

Disease status at the time of transplantation was another critical determinant of outcomes. Yanada et al. [18] found that patients transplanted in CR achieved a five-year OS of 58%, whereas those with non-CR status or prior autologous HSCT fared significantly worse (OS: 6%). These results reinforce the consensus that allo-HSCT should ideally be performed in CR, as active disease at transplantation is associated with higher relapse and inferior survival [26]. The study also identified prior autologous HSCT as an adverse prognostic factor, likely due to cumulative treatment-related toxicities and clonal evolution. These findings are corroborated by registry data showing that salvage allo-HSCT after autologous HSCT failure yields dismal outcomes, emphasizing the importance of sequencing therapies judiciously [27].

The role of allo-HSCT in relapsed APL was explored by Min et al. [19] and Sanz et al. [20], with divergent findings. While Min et al. [19] reported favorable outcomes for allo-HSCT as salvage therapy in second relapse (6/7 patients surviving), Sanz et al. [20] observed superior two-year OS with autologous HSCT (82.4% vs. 64.3%) due to lower NRM (2.7% vs. 17.3%). These discrepancies may reflect differences in patient selection, disease biology, and treatment protocols. For instance, Sanz et al. [20] included patients in second CR (CR2), whereas Min et al. [19] focused on refractory cases. The superior outcomes with autologous HSCT in CR2 align with guidelines recommending auto-HSCT for APL in molecular remission [28], while allo-HSCT remains reserved for high-risk or multiply relapsed disease.

ELN risk stratification further refined the prognostic landscape, with Socie et al. [16] demonstrating that allo-HSCT improved OS in intermediate/poor-risk AML (HR=0.51) but offered limited benefit in good-risk disease. This finding is consistent with recent European LeukemiaNet (ELN) recommendations advocating risk-adapted post-remission strategies [29]. However, the study also highlighted barriers to transplantation in older and comorbid patients, underscoring the need for innovative approaches to expand access, such as haploidentical donor protocols and reduced-toxicity conditioning [30].

The risk of bias assessment revealed generally robust methodological quality among included studies, with the RCT by Niederwieser et al. [14] and the propensity-matched cohort by Li et al. [12] exhibiting minimal bias. However, retrospective registry studies [15,17,18,20] were susceptible to unmeasured confounders, such as center-specific practices and incomplete data on conditioning regimens. The high-risk bias ratings for Min et al. [19] and Kulkarni et al. [21] reflect their small sample sizes and lack of control groups, limiting the generalizability of their findings. These limitations underscore the need for prospective, multicenter studies to validate observational data.

Limitations

Despite its comprehensive scope, this review has several limitations. Firstly, the predominance of retrospective studies introduces potential selection and information biases. Second, heterogeneity in patient populations, conditioning regimens, and outcome definitions precluded meta-analysis, limiting the ability to quantify pooled effects. Third, follow-up durations varied widely, with some studies reporting only short-term outcomes [12,17], while others provided longer-term data [14,18]. Finally, the exclusion of non-English studies may have introduced language-related bias.

## Conclusions

This review consolidates evidence supporting allo-HSCT as a curative option for adult AML, particularly in intermediate/poor-risk disease and older patients with suitable donors. Key determinants of success include donor type, conditioning intensity, disease status, and risk stratification, with MSD and CR transplantation associated with the best outcomes. Haploidentical HSCT emerges as a promising alternative for older patients, while auto-HSCT remains preferred for APL in molecular remission. Future research should prioritize prospective trials to optimize donor selection, conditioning protocols, and supportive care strategies, with the ultimate goal of expanding access and improving survival for all AML patients.

## Appendices

**Table 6:** Search strings

Database	Search string
PubMed	("acute myeloid leukemia"[MeSH Terms] OR "acute myeloid leukemia"[tiab] OR "AML"[tiab]) AND ("hematopoietic stem cell transplantation"[MeSH Terms] OR "allogeneic hematopoietic stem cell transplantation"[tiab] OR "allo-HSCT"[tiab] OR "allogeneic transplant"[tiab]) AND ("survival"[tiab] OR "outcomes"[tiab] OR "relapse"[tiab] OR "non-relapse mortality"[tiab] OR "overall survival"[tiab] OR "disease-free survival"[tiab]) AND ("2020/01/01"[Date - Publication] : "2025/08/31"[Date - Publication])
Scopus	TITLE-ABS-KEY ( "acute myeloid leukemia" OR AML ) AND TITLE-ABS-KEY ( "allogeneic hematopoietic stem cell transplantation" OR "allo-HSCT" OR "allogeneic transplant" ) AND TITLE-ABS-KEY ( survival OR outcomes OR relapse OR "non-relapse mortality" OR "overall survival" OR "disease-free survival" ) AND PUBYEAR > 2019 AND PUBYEAR < 2026
Web of Science	TS=("acute myeloid leukemia" OR AML) AND TS=("allogeneic hematopoietic stem cell transplantation" OR "allo-HSCT" OR "allogeneic transplant") AND TS=(survival OR outcomes OR relapse OR "non-relapse mortality" OR "overall survival" OR "disease-free survival") AND PY=(2020-2025)
Embase	('acute myeloid leukemia'/exp OR 'acute myeloid leukemia':ti,ab OR AML:ti,ab) AND ('allogeneic hematopoietic stem cell transplantation'/exp OR 'allogeneic hematopoietic stem cell transplantation':ti,ab OR 'allo-HSCT':ti,ab OR 'allogeneic transplant':ti,ab) AND ('survival'/exp OR survival:ti,ab OR outcomes:ti,ab OR relapse:ti,ab OR 'non relapse mortality':ti,ab OR 'overall survival':ti,ab OR 'disease free survival':ti,ab) AND (2020-2025)/py
ClinicalTrials.gov	Condition or disease: "acute myeloid leukemia" AND Other terms: ("allogeneic stem cell transplantation" OR "allo-HSCT" OR "allogeneic transplant") AND Outcome Measures: survival OR relapse OR "non-relapse mortality" OR "disease-free survival" AND Study Start: 01/01/2020 to 08/31/2025
